# Euclidean consistency-driven dual-layer information fusion framework for UAV-based traffic accident scene reconstruction

**DOI:** 10.1371/journal.pone.0350987

**Published:** 2026-06-24

**Authors:** Zhihao Xie, Wenjing Xia, Cheng He, Taifeng Qiu

**Affiliations:** 1 College of Civil Engineering, Nanjing forestry University, Nanjing, China; 2 Jiangsu Highway Intelligent Detection and Low-Carbon Maintenance Engineering Research Center, Nanjing Forestry University, Nanjing, Jiangsu, China; 3 Department of Civil and Environmental Engineering, National University of Singapore, Singapore, Singapore; 4 Investigation College, Nanjing Police College, Nanjing, Jiangsu, China; Xidian University, CHINA

## Abstract

This study aims to develop an interpretable dual-layer information fusion framework driven by the Euclidean Consistency Index (ECI) for UAV flight parameter optimization, which integrates a statistically interpretable linear module with a nonlinear learning layer implemented via support vector regression to enhance 3D reconstruction accuracy in traffic accident scenarios. Experiments based on UAV oblique photogrammetry (27 configurations) were conducted to evaluate reconstruction accuracy using elevation error, horizontal error, and distortion anomaly metrics. The results reveal that flight altitude and image overlap exert coupled effects on reconstruction performance. An optimal parameter range is identified as 20–25 m altitude, 80–85% forward overlap, and 70–75% side overlap, achieving centimeter-level accuracy and stable geometric consistency. The high inter-layer agreement (ECI = 0.9133) confirms the robustness of the proposed fusion strategy. Validation in a real-world accident scene offers preliminary support for its practical applicability under similar conditions. Within the tested experimental scope, this framework provides a quantitatively interpretable and operationally stable foundation for UAV flight parameter selection in accident reconstruction.

## Introduction

With the rapid development of Unmanned Aerial Vehicles (UAV) technology, there has been a significant increase in applied research in traffic accident scene investigation [1,2]. Three-dimensional (3D) reconstruction of traffic accident scenes has been recognized as highly valuable for causation analysis, scene reconstruction, and legal evidence collection [3,4]. Conventional survey methodologies predominantly relied on manual measurement and two-dimensional photographic records. However, these methods had many limitations, including reduced data collection efficiency, lack of spatial information, and increased risk of secondary accidents [5–8]. In recent years, UAV oblique photogrammetry was established as a critical tool for traffic accident investigation, owing to high-resolution multi-angle imaging and rapid deployment capabilities of the technology [7]. This technological advancement substantially enhanced both the accuracy and efficiency of 3D accident scene reconstruction [9].

In recent years, UAV oblique photogrammetry has advanced significantly in the field of 3D reconstruction for traffic accident investigations. The mechanisms through which flight parameters influence modeling accuracy have been systematically explored. Flight altitude has been demonstrated to affect accuracy via ground sampling distance (GSD) and geometric distortion. Flights conducted at low altitudes, specifically in the range of 15–30 meters, enhanced resolution but exacerbated nonlinear distortions [10,11]. A dynamic height adjustment strategy was proposed by Fraser and Congalton, reducing vertical errors to 12 cm through synergistic optimization of height and structure from motion parameters [12]. For traffic accidents, dynamic height with centimeter-level imagery was validated for fine-grained modeling [13], while height-terrain matching lowered digital terrain model errors by 35% in low-control-point scenarios [14].

The nonlinear impact of forward overlap on matching robustness was widely investigated. Previous research reported that optimized forward overlap reduced errors by 15–20% in traffic scenarios. In forestry studies, over 95% overlap suppressed canopy errors by 15% [11,15]. In contrast, achieving sub-decimeter vertical accuracy was found to sometimes require dynamic overlap adjustments [16]. Meanwhile, agricultural research determined that combining 95% overlap with a 100‑meter flight altitude provided an optimal cost‑benefit ratio [17].

Previous studies critically examined the role of side overlap in ensuring inter‑strip continuity [18,19]. For instance, one study reported that an 85% side overlap improved model completeness by 12% [19]. In contrast, researchers showed that insufficient overlap induced discrepancies of up to 20 cm in digital surface models [10]. Existing research in this area predominantly relied on static image analysis. The potential of video streaming with very high overlap rates remained largely unexplored. However, a later study validated the use of UAV video frames, with overlaps between 82.5% and 97.5%, for rapid non‑invasive mapping [15]. That approach achieved a root mean square error of 5–8 cm, outperforming traditional tape measurements. Nevertheless, the application of this method for modeling dynamic objects still required further verification.

Previous studies investigated multi-parameter interactions and identified image overlap and ground control point quantity as the dominant factors, which exhibited nonlinear effects on accuracy [20]. One prior study showed that low-altitude flights between 15 and 30 m, combined with 99% forward overlap, enhanced reconstruction details. The same study found that a side overlap of 50–70% balanced efficiency with model completeness [21]. Another study reported that flights at a 5-meter altitude using a point-of-interest technique achieved optimal accuracy [22]. Furthermore, research demonstrated that low-altitude flights with high overlap (over 70%) significantly improved both horizontal and vertical accuracy. Centimeter-level root mean square error was attained under such configurations [23].

Extensive studies were systematically reviewed to evaluate advancements in flight parameter optimization for UAV tilt-photography modeling. Multi-parameter co-optimization strategies proved successful in forestry, agriculture, and other related disciplines, yet these existing configurations lacked the precision required for traffic accident reconstruction scenarios. Although preliminary efforts adapted multi‑parameter co‑optimization techniques to traffic accident investigations, a unified framework was seldom established. Such a framework would concurrently optimize three critical parameters: flight altitude, forward overlap, and side overlap.

This study introduces a Euclidean Consistency-Driven Dual-Layer Information Fusion Framework for UAV-based traffic accident scene reconstruction. A key innovation is the proposal of distortion anomaly as a novel model quality metric. The framework consists of a statistically interpretable linear layer and an adaptive nonlinear layer, integrated through a newly proposed Euclidean Consistency Index (ECI). The ECI dynamically balances the contributions of both layers, bridging the gap between linear and nonlinear modeling. This establishes a robust and interpretable paradigm for optimizing UAV photogrammetry in forensic applications.

## Materials and methods

### Experimental design

The experiment simulated a vehicle side-swipe scenario on Majiayuan Road in Qixia District, Nanjing, China. Data were collected under clear sky conditions at an ambient temperature of 16 °C with a steady southeast wind of 2 m/s. The UAV imaging system used a DJI Matrice 350 RTK platform, which was equipped with a Zenmuse P1 full-frame camera (35 mm focal length) and a D‑RTK2 mobile positioning station. Camera calibration was performed prior to data acquisition using the built-in calibration procedure of the Zenmuse P1 camera. The in-flight calibration was performed at 102 m altitude with the camera pointing nadir (−90°). Feature-rich ground areas were selected to guarantee reliable calibration, following DJI’s standard protocol. Ground truth measurements came from an NTS-332R10M non-prism total station, with an accuracy of ± (2 mm + 2 × 10^−6^ D), and a certified Grade I engineering tape. Baseline flight parameters included a flight altitude of 25 m, forward overlap of 80%, and side overlap of 70%. These parameters were chosen based on actual urban road traffic conditions and standard UAV photogrammetry practices to ensure sufficient image coverage and high-quality reconstruction. The gimbal tilt was fixed at 45 ° with automatic attitude stabilization. Other parameters were optimized autonomously. A univariate gradient adjustment was applied to three key parameters. The flight altitude was defined as ranging from 20 to 80 m. The forward overlap ranged from 60% to 90%, and the side overlap ranged from 45% to 85%.

### Model construction, data acquisition and analysis

**Fig 1:**
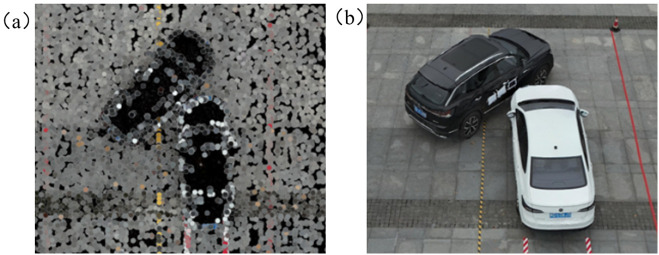
3D reconstruction of a road traffic accident scene.(a) Modeling point clouds, (b) Texture mapping model.

The 3D reconstruction was performed using DJI Terra (v5.1.0), and the World Geodetic System 1984 (WGS84) was adopted as the geographic coordinate system. The original images first underwent distortion correction and exposure adjustment to ensure consistent radiometric measurement quality. Aerial triangulation was then performed using feature matching and bundle adjustment algorithms, through which a sparse point cloud was generated and camera pose parameters were optimized. A dense point cloud was then produced using the multi-view stereo (MVS) algorithm, followed by noise filtering and topological optimization to accomplish surface reconstruction and texture mapping. Ultimately, a refined 3D mesh model with realistic illumination effects and color consistency was obtained (Fig 1). The entire workflow from image import to textured model generation was completed using the built-in automated processing pipeline of DJI Terra.

For ground truth collection, elevations and horizontal dimensions in the physical scene were measured with a prismless total station and a Grade I engineering tape. Corresponding measurements within the 3D model were obtained using the software’s built-in tools. Each object was measured three times, and the median value was adopted to minimize random error. In contrast to the mean, the median was less sensitive to extreme values, thereby reducing the impact of measurement noise or reconstruction artifacts on potential outliers. Due to the high precision of the field measuring instruments, all measured data were treated as the ground truth.

For the distortion anomaly metric, anomalies were identified and counted manually by three independent operators following a unified set of predefined criteria. Each operator performed the counting separately, and the final anomaly count was determined as the median of the three observations to ensure robustness and reduce subjective bias. Inter-rater reliability was assessed using the intraclass correlation coefficient (ICC).

All statistical analyses, including correlation analysis, weight computation, and model fusion, were performed in Python 3.13.2. The core analytical libraries used were scikit-learn (v1.7.1), numpy (v2.2.6), and scipy (v1.16.1). Data visualization was supported by Origin 2024b. The normality of the variables was assessed using the Shapiro-Wilk test. The results indicated that all reconstruction accuracy indicators deviated significantly from normality (p < 0.05). Given the limited sample size (n = 27) and the non-normal distribution characteristics, non-parametric Spearman rank correlation analysis was employed to evaluate the associations between flight parameters and reconstruction accuracy. The significance level (α) was set at 0.05 for all statistical tests. Given the exploratory nature of the analysis and the limited sample size, no formal multiple comparison correction (e.g., Bonferroni adjustment) was applied. Instead, the interpretation of results emphasized the combined consideration of statistical significance, effect size (r²), and the consistency of observed trends to reduce the risk of Type II errors associated with overly conservative corrections.

### Quantitative metrics for model accuracy assessment

**Fig 2:**
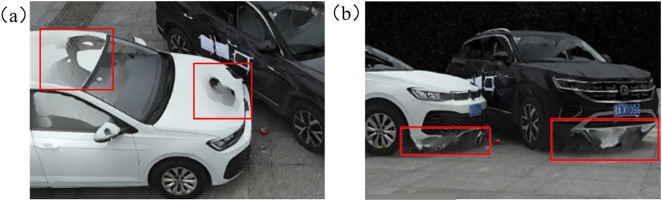
Examples of distortion anomalies diagram. **(a)** Void distortion, defined as missing surface regions in the reconstructed model that are visibly discontinuous from the surrounding geometric structure and form observable gaps (e.g., incomplete reconstruction of vehicle roofs or hoods). **(b)** Stretching distortion, defined as abnormal geometric elongation or artificial connections between structures that should be spatially separated. This phenomenon commonly occurred in the transition region between the vehicle chassis and the ground due to insufficient image coverage caused by imaging angle limitations.

The accuracy of the 3D reconstruction model was evaluated using three core metrics: elevation error, horizontal error, and distortion anomaly. These metrics collectively reflected both geometric and non‑geometric errors in accident scene reconstruction [20,24]. Among them, elevation error was used to assess vertical accuracy and directly determined the reconstruction accuracy of road surfaces and object heights, thereby serving as a key evaluation basis for traffic accident analysis. Horizontal error quantified positional deviation in the plane. This measurement was essential for accurately locating vehicles, road markings, and other scene elements. Distortion anomaly served as a complementary metric to capture reconstruction defects not reflected by geometric errors. The operational definition of this indicator was visually identifiable abnormal defects in the reconstructed model, which were specifically classified into two categories: (a) void distortion, characterized by missing surface regions that were discontinuous from the surrounding geometry, and (b) stretching distortion, characterized by abnormal geometric elongation or artificial connections between spatially independent structures (Fig 2). This metric was quantified by counting the number of spatially distinct distorted regions in the model. This complementary assessment of model quality was particularly crucial in forensic applications due to its direct influence on the reliability of evidentiary data.

### Weighted modeling and correlation constraint mechanism in the linear information layer

To ensure statistical interpretability and mitigate redundancy among error metrics, the linear information layer integrated entropy weighting, Pearson-based correlation correction, and the CRITIC model within a unified Euclidean Consistency framework. This design allowed for a balanced representation of information disparity, inter-metric independence, and conflict intensity.

First, information entropy quantified the effective dispersion of each error metric, offering an initial estimate of the informational contribution from each metric. Lower entropy indicated higher variability, which led to a greater initial weight. However, correlated metrics could introduce redundant information. Therefore, we used the Pearson correlation matrix to adjust each entropy weight based on its average interdependence. This adjustment reduced the weight of indicators with high linear redundancy to align their contribution with independent statistical significance.

Next, the CRITIC model refined the objective importance of each metric. This refinement combined information variability, represented by standard deviation, with conflict intensity, measured by correlation strength. Metrics with high variability and strong differentiation from others received higher CRITIC weights. This captured structural contrast within the data set. To harmonize the statistical sensitivity of entropy weighting and the structural independence of CRITIC, we introduced the ECI as a geometric regulator:


$$\begin{array}{c} \alpha =ECI\text{(}\omega^{\left(EP\right)},\omega^{\left(C\right)}\text{)}=1-\frac{{\|\omega^{\left(EP\right)}-\omega^{\left(C\right)}\|}_{\text{2}}}{{\|\omega^{\left(EP\right)}\|}_{\text{2}}+{\|\omega^{\left(C\right)}\|}_{\text{2}}} \end{array}$$ (1)

where ||·||_2_ denoted the Euclidean norm. The normalized weight vectors from the entropy-weight-Pearson and CRITIC schemes were represented as $\omega^{\left(EP\right)}$ and $\omega^{\left(C\right)}$, respectively. The ECI was defined to measure the spatial agreement between these two weighting schemes. Its value lied in the interval [0, 1], where a larger value indicated stronger consistency between the weighting strategies.

The final fused weight vector for the linear layer was calculated as:


$$\begin{array}{c} W^{\left(L\right)}=\alpha \omega^{\left(EP\right)}+(1-\alpha )\omega^{\left(C\right)} \end{array}$$ (2)

This fusion dynamically balanced information disparity and conflict intensity under a geometric constraint. This balance enhanced interpretability while maintaining sensitivity to parameter-driven variability. The resulting linear layer weight field provided a statistically coherent foundation for subsequent nonlinear learning and cross-layer fusion.

Consequently, the resulting linear layer weight field provided a statistically coherent foundation for subsequent nonlinear learning and cross-layer fusion.

### Weight learning in the nonlinear feature layer

To model the complex nonlinear relationships between flight parameters and reconstruction errors, this study employed Support Vector Regression (SVR). This method served as the nonlinear feature layer. The SVR design followed the Structural Risk Minimization principle, which minimized a bound on generalization error rather than only the training data error. Consequently, SVR demonstrated particular robustness under the limited, correlated data conditions common in UAV photogrammetry [25]. The SVR offered greater stability with small sample sizes compared to methods like Random Forests or Neural Networks. The Radial Basis Function (RBF) kernel was used. This kernel not only enabled efficient nonlinear mapping, but also helped preserve model smoothness and interpretability. Therefore, SVR struck an appropriate balance among adaptability, computational efficiency, and model transparency.

The model input features included flight altitude, forward overlap, and side overlap, while the output variables comprised three indicators of 3D reconstruction quality: elevation error, horizontal error, and distortion anomaly. Each output variable was independently modeled as a continuous response variable. Prior to model training, all input features were preprocessed using the Z-score standardization method to ensure comparability of feature scales and thereby improve model stability. A fixed random seed (random state = 42) was set during the cross-validation procedure to ensure reproducibility.

The SVR optimization problem was formulated as minimizing the regularized empirical risk:


$$\begin{array}{c} \underset{\omega ,b,\xi_{i},\overset{*}{\underset{i}{\xi }},}{min}\frac{\text{1}}{\text{2}}{\|\omega \|}_{\text{2}}+C\sum_{i=1}^{n}(\xi_{i}+\overset{*}{\underset{i}{\xi }}) \end{array}$$ (3)

subjected to the ε-insensitive constraints:


$$\begin{array}{c} \left\{ \begin{array}{c} @l@y_{i}-(\omega \cdot \varphi (x_{i})+b)\leq \varepsilon +\xi_{i},\\ (\omega \cdot \varphi (x_{i})+b)-y_{i}\leq \varepsilon +\overset{*}{\underset{i}{\xi }},\\ \xi_{i},\overset{*}{\underset{i}{\xi }}\geq 0 \end{array}\right. \end{array}$$ (4)

where ω denoted the regression coefficient vector and b represents the bias term. The function $\emptyset ({\text{x}}_{\text{i}})$ mapped the input vector ${\text{x}}_{\text{i}}$ from the original feature space to a higher-dimensional kernel space. The slack variables $\xi_{\text{i}}$ and $\overset{*}{\underset{\text{i}}{\xi }}$ measured deviations outside the epsilon-insensitive margin. The parameter C controlled the trade-off between model flatness and empirical error, while ε defined the width of the epsilon-insensitive loss zone. The kernel scale parameter γ determined the sensitivity of the Radial Basis Function (RBF) kernel, defined as $\text{K}({\text{x}}_{\text{i}},{\text{x}}_{\text{j}})=\text{exp}(-\gamma {\left\|{\text{x}}_{\text{i}}-{\text{x}}_{\text{j}}\right\|}^{\text{2}})$.

To ensure the reproducibility of the results, this study optimized the SVR hyperparameters (C, ε, γ) using a constrained grid search combined with five-fold cross-validation. A fixed random seed (random state = 42) was applied in the cross-validation procedure. The hyperparameter search space was defined as follows: C ∈ {1, 10, 50}, ε ∈ {0.01, 0.1, 0.2}, and γ ∈ {‘scale’, 0.01}, where ‘scale’ set γ as the inverse of the product of feature number and feature variance. The SVR model in this study adopted the default convergence tolerance (tol = 0.001) in scikit-learn library. Given the small dataset size (n = 27), the grid search procedure required only a few minutes on a standard workstation. The nonlinear weight vector ${\text{W}}_{\left(\text{N}\right)}$ was determined from the average cross-validated coefficient of determination $\overset{\text{2}}{\underset{\text{CV},\text{j}}{\text{R}}}$ for each output metric:


$$\begin{array}{c} \overset{\left(N\right)}{\underset{k}{W}}=\frac{\overset{\text{2}}{\underset{CV,k}{R}}}{\overset{m}{\underset{j=1}{\sum }}\overset{\text{2}}{\underset{CV,j}{R}}},k=1,2,...m \end{array}$$ (5)

where m = 3 in this study. The resulting normalized weights quantified the relative nonlinear contributions of each error metric, providing the foundation for subsequent Euclidean-Consistency-based fusion.

### Adaptive fusion strategy and comprehensive error function

To adaptively coordinate the linear and nonlinear layers, we introduced the ECI as a fusion regulator. The ECI dynamically balanced the influence of the two layers according to their weight differences, enabling consistency-aware cross-layer fusion. This strategy supported self-regulated weight adjustment through inter-layer constraints and established a global accuracy model via a comprehensive error function.

In this dual-layer framework, the linear layer ensured statistical stability and interpretability, while the nonlinear layer captured adaptive learning patterns. The structural difference between their weight vectors was measured by the ECI, which served as the fusion coefficient for computing the integrated weights, as defined in Equations (6) and (7).


$$\begin{array}{c} \lambda =ECI\text{(}W^{\left(L\right)},W^{\left(N\right)}\text{)}=1-\frac{{\|W^{\left(L\right)}-W^{\left(N\right)}\|}_{\text{2}}}{{\|W^{\left(L\right)}\|}_{\text{2}}+{\|W^{\left(N\right)}\|}_{\text{2}}} \end{array}$$ (6)

$$\begin{array}{c} W^{*}=\lambda W^{\text{(}L\text{)}}+(1-\lambda )W^{\text{(}N\text{)}} \end{array}$$ (7)

The parameter λ = ECI regulated the contribution ratio of the two layers, with ECI ranging between 0 and 1. When ECI was high, the model prioritized linear statistical features. When ECI was low, the model increased the weight of the nonlinear layer. This compensatory fusion maintained a balance between stability and interpretability across different consistency intervals.

To unify accuracy evaluation across metrics, a comprehensive error function was defined in Equation (8).


$$\begin{array}{c} F\left(ECI\right)=\sum_{j=1}^{n}W^{*}\cdot p_{ij} \end{array}$$ (8)

Where ${\text{p}}_{\text{ij}}\;$ denoted the standardized value of the j-th error metric for the i-th configuration.

### Ethics statement

All UAV flight operations and field surveys are conducted with prior authorization and in coordination with local traffic police authorities. The real-world traffic accident scene used in this study is documented as part of an officially permitted investigation process. No identifiable personal information (e.g., faces, license plates, or private property details) is collected during data acquisition. Therefore, no anonymization or redaction procedures are required. This study does not involve human subjects or personal data and therefore does not require formal ethical approval. All procedures are conducted in accordance with relevant regulations and research integrity standards for field-based studies.

## Results and analysis

### Analysis of flight parameters and error distribution characteristics

**Fig 3:**
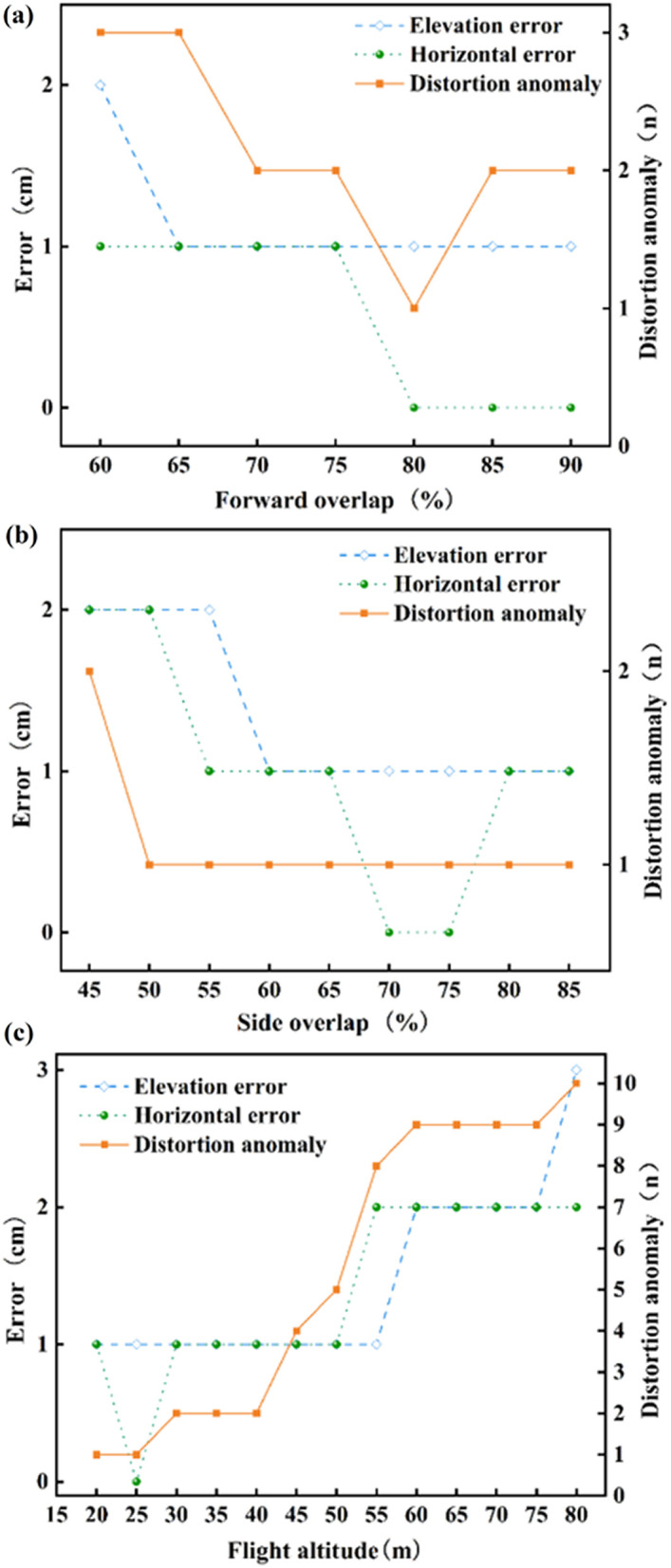
Influence of flight parameters on error metrics. (a) forward overlap, (b)side overlap, and (c) flight altitude.

Fig 3 showed the influence of flight altitude, forward overlap, and side overlap on reconstruction accuracy. Table 1 provided the corresponding Spearman correlation analysis. Together, they illustrated how each parameter distinctly affects geometric consistency and photogrammetric precision, while revealing their interdependent effects.

**Table 1:** Spearman correlation analysis between flight parameters and error metrics.

Flight Parameter	Error Metric	Correlation Coefficient (ρ)	p-value	r^2^	Significance
Forward Overlap	Elevation Error	−0.612	0.144	0.37	ns
	Horizontal Error	−0.866	0.012	0.75	*
	Distortion Anomaly	−0.697	0.082	0.49	ns
Side Overlap	Elevation Error	−0.822	0.007	0.68	**
	Horizontal Error	−0.645	0.060	0.42	ns
	Distortion Anomaly	−0.548	0.127	0.30	ns
Flight Altitude	Elevation Error	0.861	<0.001	0.74	***
	Horizontal Error	0.877	<0.001	0.77	***
	Distortion Anomaly	0.979	<0.001	0.96	***

Table note: ρ = Spearman’s rank correlation coefficient; ns = not significant (p ≥ 0.05); * p < 0.05, ** p < 0.01, *** p < 0.001. The effect size (r²) is calculated as the squared correlation coefficient to indicate the strength of the relationship.

Forward overlap improved both elevation and horizontal accuracy when increased from 60% to 85%. Elevation error decreased from about 2 cm to 1 cm. Horizontal error dropped from 1 cm to nearly 0 cm. Distortion anomalies were reduced from 3 to 1. Correlation analysis showed a strong negative correlation between forward overlap and horizontal error (ρ = −0.866, p = 0.012). This indicated that higher image redundancy enhanced feature matching and stabilizes bundle adjustment. However, accuracy gained diminished beyond 85% forward overlap, suggesting a saturation threshold.

Side overlap evidently improved vertical reconstruction accuracy. As side overlap increased from 45% to 80%, the elevation error decreased from 2 cm to 1 cm, and a statistically significant negative correlation was observed between the two variables (ρ = −0.822, p = 0.0066). This improvement was attributed to enhanced stereo-intersection geometry and strengthened the consistency of observations in the vertical dimension. In contrast, horizontal error and distortion anomaly exhibited negative associations with increasing side overlap (ρ = −0.645, p = 0.060; ρ = −0.548, p = 0.127), although these relationships did not reach statistical significance. This suggested that the influence of side overlap on planar accuracy and distortion anomalies was comparatively weaker and less robust than its effect on vertical reconstruction accuracy.

Flight altitude negatively affected all error metrics. As flight altitude increased from 20 m to 80 m, elevation error grew from 1 cm to 3 cm. Horizontal error increased from 1 cm to 2 cm. Distortion anomalies rose sharply from 1 to about 10. All correlations with altitude were strongly positive (ρ = 0.861–0.979, p < 0.001). Higher altitude enlarged GSD, weakened stereo parallax, and reduced feature detectability, thereby amplifying error propagation.

Overall, forward overlap primarily governed planar accuracy, side overlap determined vertical consistency, and flight altitude dominated scale-dependent error propagation. These statistically validated trends confirm that the Euclidean-Consistency-driven fusion model accurately captured the coupled relationships among image redundancy, parallax geometry, and reconstruction reliability. This provided a consistent theoretical foundation for optimizing UAV flight parameters.

### Error metric statistics and linear layer analysis

Before linear coupling analysis, inter‑rater reliability of the distortion anomaly metric was assessed using three independent operators’ counts from 27 unique configurations. The intraclass correlation coefficient (ICC) was 0.964 (95% CI: 0.930–0.980), indicating excellent agreement. This confirms the metric’s reproducibility for subsequent linear and nonlinear modeling with elevation and horizontal errors.

To elucidate the linear coupling relationships among multiple error metrics and their impact on UAV aerial survey parameter optimization, this study constructed a linear information layer weighting system based on the “Entropy Weight-CRITIC-ECI” framework. This system aimed to comprehensively reflect the informational disparity, structural independence, and linear correlations among the error metrics. The entropy weight method was used to measure the statistical variability of each metric. The CRITIC model integrated both the standard deviation and the linear correlation coefficients to characterize the conflict intensity between metrics. The ECI was then employed to harmonize the geometric consistency between these two linear weighting schemes, enabling optimized weight fusion. Table 2 presented the entropy weights, CRITIC weights, and ECI-fused resulted for each error metric. Table 3 displayed the Pearson correlation coefficient matrix among the error metrics.

**Table 2:** Weight distribution in the linear layer.

Error Metric	Entropy Weight	CRITIC Weight	Linear Layer Weight
Elevation Error	0.1296	0.3008	0.1755
Horizontal Error	0.6085	0.3368	0.5356
Distortion Anomaly	0.2619	0.3625	0.2889

**Table 3:** Pearson correlation coefficient matrix.

Error Metric	Elevation Error	Horizontal Error	Distortion Anomaly
Elevation Error	1.0000	0.6714	0.6196
Horizontal Error	0.6714	1.0000	0.6899
Distortion Anomaly	0.6196	0.6899	1.0000

The results revealed clear differences between the entropy weight method and the CRITIC model. Under the entropy method, horizontal error received the highest weight. The assigned value was 0.6085, which was significantly greater than the weight for elevation error at 0.1296 and that for distortion anomaly at 0.2619. This weighting outcome stemmed from the greatest fluctuation by horizontal error in the sample space. Consequently, horizontal error contributed the most information under the disparity-based framework, confirming its dominant role in geometric precision through superior sensitivity to flight parameter changes. Conversely, elevation error exhibited smaller fluctuations (σ = 0.2712), resulting in a lower weight and suggesting a more stable distribution.

The CRITIC model, which accounted for inter‑metric correlation, produced a more balanced weight distribution. It used standard deviation and conflict intensity ($\text{1}-{\text{r}}_{\text{ij}}$) to assess each metric. Elevation error exhibited the lowest average correlation with other metrics. The mean correlation coefficient was 0.645, indicating stronger linear independence. Consequently, its conflict information quantity ${\text{C}}_{\text{j}}$ increased, raising its CRITIC weight to 0.3008. Conversely, horizontal error and distortion anomaly showed high mutual correlation. The Pearson correlation coefficient between them was 0.6899, indicating substantial information redundancy. Their weights were therefore suppressed in the CRITIC model. In summary, the CRITIC model considered both variability and redundancy, better reflecting each metric’s independent contribution.

To combine both approaches, the ECI was introduced to fuse the entropy and CRITIC weights geometrically. The ECI value was 0.7316, showing moderate consistency between the two methods. The fused linear‑layer weights ${\text{W}}_{\left(\text{L}\right)}$ balanced the sensitivity of entropy weighting with the stability of CRITIC. Horizontal error maintained its dominant position with a final weight of 0.5356, although the margin over other metrics narrowed. Elevation error increased to a weight of 0.1755, while the distortion anomaly maintained a moderate weight of 0.2889. This outcome showed the ECI fusion reduced the entropy method’s oversensitivity to variable metrics, achieving a better balance between discriminability and equilibrium.

In summary, the linear-layer weights reflected key error metric characteristics. Horizontal error held the highest weight, indicating strong parameter sensitivity and a dominant role in planar accuracy. Elevation error exhibited the lowest inter-metric correlation, reflecting its independent influence on vertical accuracy. Occupying an intermediate position, the distortion anomaly modulated the effect of image texture complexity on geometric consistency. The fused linear layer balanced interpretability with structural stability, providing a robust prior for subsequent nonlinear learning and dual-layer information fusion.

### Construction of the nonlinear information layer and analysis of results

The RBF-SVR model effectively captured the nonlinear relationships between UAV flight parameters and 3D reconstruction errors. All error metrics achieved high fitting accuracy in training ($\overset{\text{2}}{\underset{\text{full}}{\text{R}}}\;>\;\text{0}.\text{8}$), confirming the model’s ability to represent complex input-output dependencies.

Horizontal error showed the highest nonlinear stability in cross‑validation ($\overset{\text{2}}{\underset{\text{CV}}{\text{R}}}$ = 0.51 ± 0.19). This metric received the largest weight. The assigned value was 0.4909, demonstrating strong sensitivity to overlap variation. Higher forward and side overlap increased matching redundancy, which improved bundle adjustment stability. In the RBF kernel space, this redundancy appeared as higher similarity between samples, allowing the model to capture the nonlinear reinforcement of feature consistency.

Elevation error was mainly influenced by flight altitude ($\overset{\text{2}}{\underset{\text{CV}}{\text{R}}}$ = 0.31 ± 0.36 weight = 0.2991). As altitude increased, ground sampling distance grew and the stereo intersection angle decreased. This amplified vertical triangulation error. The RBF kernel mapped this gradual error amplification effectively. Distortion anomaly showed high local variance ($\overset{\text{2}}{\underset{\text{CV}}{\text{R}}}$ = 0.21 ± 1.34), reflecting its sensitivity to texture irregularities and parallax inconsistency. Its weight was 0.2100.

**Fig 4:**
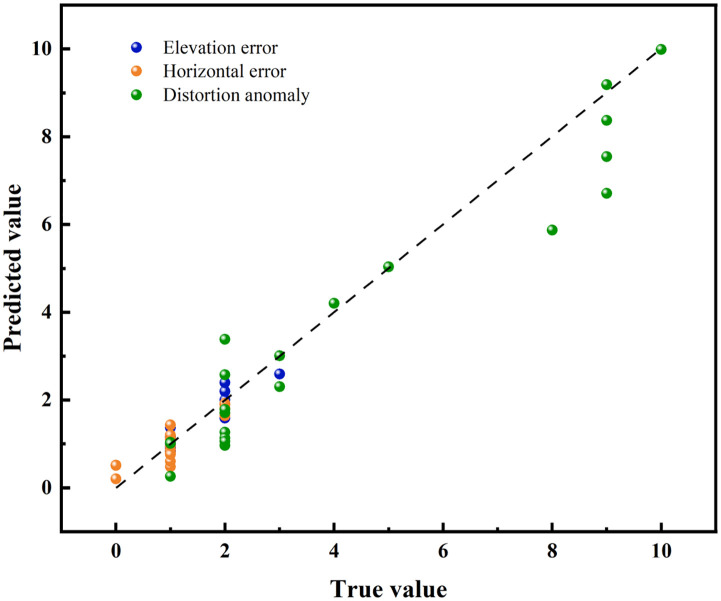
SVR fitting effect plot.

To combine both approaches, we introduced the ECI to fuse the entropy and CRITIC weights geometrically. The weight for elevation error was 0.2991, for horizontal error was 0.4909, and for the distortion anomaly was 0.2100. Horizontal accuracy acted as the primary nonlinear driver. Elevation error and distortion anomaly provided secondary nonlinear contributions. Scatter plots aligned closely along the 1:1 reference line between predictions and observations. This confirmed both local and global model stability (Fig 4).

To assess model robustness against data partitioning and to empirically justify the choice of the nonlinear component, we compared the SVR-based nonlinear layer with a second-degree polynomial regression baseline using nested repeated cross-validation (5 × 5 folds, 25 evaluations) with inner grid search for SVR. Table 4 summarizes the cross-validated R^2^ for each error metric for both models.

**Table 4:** Repeated nested cross-validation performance (5 × 5 folds) of SVR and polynomial regression (degree = 2).

Model	Error Metric	Mean R^2^	Std	95%CI
SVR	Elevation Error	0.403	0.298	[−0.270, 0.804]
	Horizontal Error	0.426	0.273	[−0.109, 0.774]
	Distortion Anomaly	0.138	1.922	[−4.730, 0.972]
Polynomial	Elevation Error	0.329	0.426	[−0.323, 0.822]
	Horizontal Error	0.313	1.079	[−2.035, 0.803]
	Distortion Anomaly	0.689	0.553	[−0.933, 0.968]

The SVR-based layer achieves moderate and stable predictive performance for elevation and horizontal errors. The mean R^2^ values are 0.403 and 0.426, with standard deviations of 0.298 and 0.273, respectively. In contrast, polynomial regression yields lower mean R^2^ (0.329 and 0.313) and substantially higher variances (0.426 and 1.079). This indicates that SVR generalizes better and is less prone to overfitting under small-sample conditions.

For distortion anomaly, polynomial regression shows a higher mean R^2^ (0.689) but also a large standard deviation (0.553). However, this metric has the lowest weight in the subsequent fusion framework, reflecting its secondary importance. The high variance for distortion anomaly in both models reflects the inherent difficulty of predicting discrete count data with a small sample size. This finding is consistent with the large standard deviation reported in the single cross-validation (0.21 ± 1.34).

Overall, the repeated resampling analysis provides empirical support that the SVR-based layer does not exhibit severe overfitting and maintains reasonable generalization under the tested conditions. The comparison with polynomial regression further justifies its selection as the nonlinear component.

### Consistency-regulated fusion weighting and flight parameter optimization

**Fig 5:**
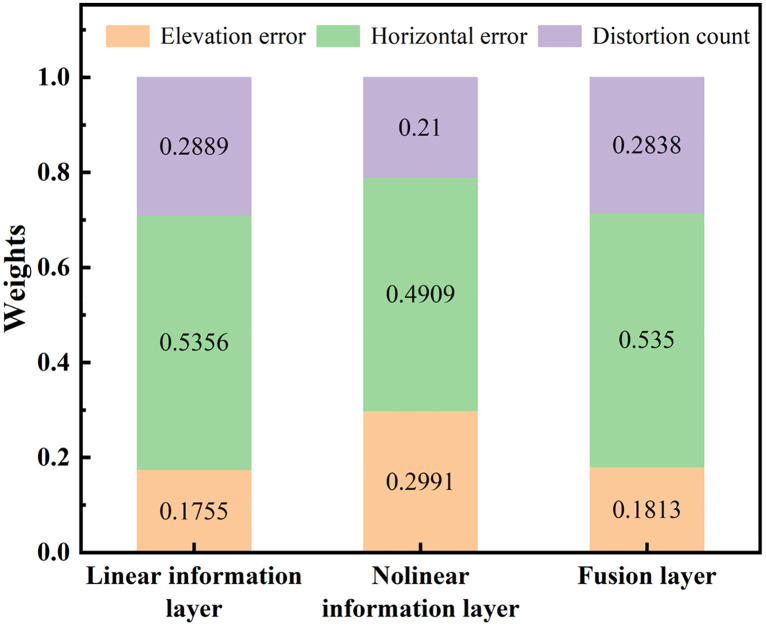
Inter-layer weight distribution plot.

We computed the ECI from the linear and nonlinear layer weights. An ECI value of 0.9133 indicated high geometric alignment between the statistical and learning components. This strong agreement implied effective mutual reinforcement between the Entropy-CRITIC linear layer and the SVR‑based nonlinear layer. The final fused weights were 0.1813 for elevation error, 0.5350 for horizontal error, and 0.2838 for distortion anomaly (Fig 5). Horizontal error held the dominant weight, confirming its role as a global sensitivity indicator for both planar accuracy and overall reconstruction precision. The weight for distortion anomaly increased relative to the linear layer, reflecting the nonlinear model’s improved ability to capture higher‑order geometric inconsistencies. Elevation error contributed steadily, showing dependence on systematic scaling rather than parameter coupling.

**Fig 6:**
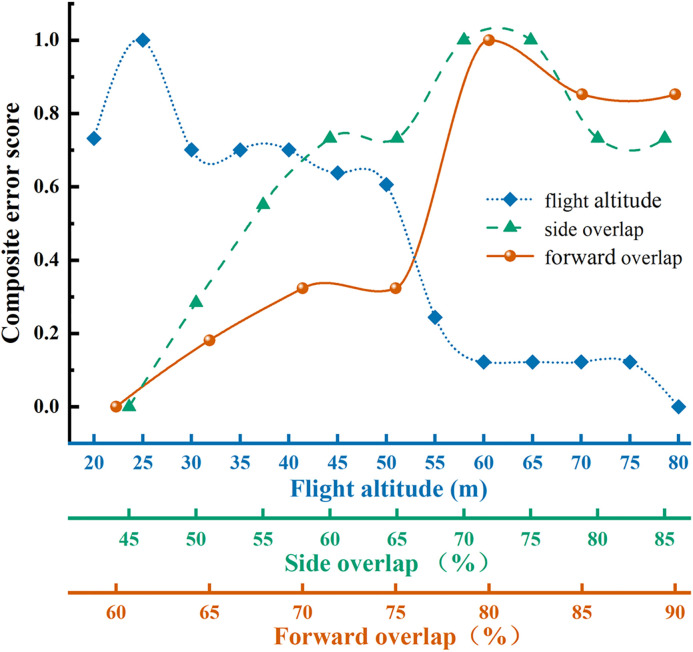
Comprehensive error score plot.

We conducted a comprehensive performance evaluation using the fused weights (Fig 6). The analysis revealed clear parametric trends. Horizontal accuracy and geometric continuity improved as image overlap increased, with convergence near 80–85%. Beyond this range, additional overlap gave diminishing returns due to information saturation. Side overlap stabilized in the 70–75% interval. Within this range, lateral continuity and computational efficiency were jointly optimized. Further overlap offered limited gains while substantially increasing data redundancy. Flight altitude showed a pronounced nonlinear effect. At 20–25 m, high‑resolution imagery and sufficient stereo parallax minimized both elevation and planar errors. Above 60 m, errors increased rapidly because of enlarged ground sampling distance and degraded feature matching.

The integrated results indicated an optimal flight configuration with a flight altitude of 20–25 m, a forward overlap of 80–85%, and a side overlap of 70–75%. This combination yielded the highest comprehensive accuracy score, indicating superior balance among vertical precision, planar consistency, and geometric stability. The high inter‑layer coherence (ECI = 0.9133) further validated the fusion framework. This enhanced interpretability while ensuring robust adaptability across varying operational conditions.

### Validation using real-world traffic accident scenes

A validation experiment was conducted at a real highway accident site in Nanping City, Fujian Province. The site was located in the Wuyi Mountain area, which featured sloped terrain, dense vegetation, and a complex road environment. The experiment aimed to test the applicability and robustness of the optimal flight parameters derived from the dual‑layer fusion model. The DJI Matrice 350 RTK UAV with a Zenmuse P1 camera was used. Four parameter sets were tested within the optimized intervals, with the flight altitude set to 20–25 m, the forward overlap to 80–85%, and the side overlap to 70–75%. The flight configurations and corresponding reconstruction errors were listed in Table 5.

**Table 5:** 3D reconstruction errors under different flight parameters.

Group	Flight Altitude (m)	Forward Overlap (%)	Side Overlap (%)	Elevation Error (cm)	Horizontal Error (cm)	Distortion anomaly (n)
1	20	80	70	1	1	1
2	20	85	75	1	1	1
3	25	80	70	1	1	2
4	25	85	75	1	1	1

The results showed centimeter‑level accuracy across all four parameter sets. Elevation and horizontal errors remained at 1 cm. The distortion anomaly count varied between 1 and 2, indicating stable geometric consistency within the optimal parameter range. Increasing forward overlap to 85% reduced the distortion anomaly to 1 in both relevant comparisons. This suggested that higher forward overlap improved image constraints and edge registration. Raising the flight altitude from 20 m to 25 m did not significantly affect positional errors, but slightly increased distortion anomaly. This was mainly due to lower ground resolution, larger blind spots, and reduced stereo intersection angles at higher altitudes, which weakened local texture constraints.

**Fig 7:**
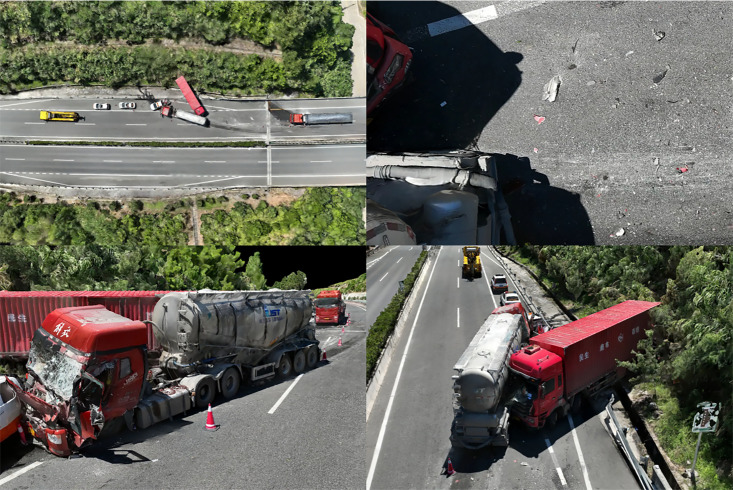
Rendering of the real-accident model.

We identified the optimal flight parameters as an altitude of 20–25 m, a forward overlap of 80–85%, and a side overlap of 70–75%. This interval achieved a good balance between accuracy and efficiency in the real accident scenario. The reconstructed 3D models showed strong spatial continuity and geometric consistency. These features were clearly preserved in detailed structures such as vehicle edges, skid marks, and scattered debris (Fig 7).

These findings confirmed the engineering feasibility and statistical robustness of the dual‑layer fusion model for real‑world traffic accident reconstruction. The model provided a quantifiable basis for standardizing UAV flight parameters and enabling rapid scene modeling in forensic applications.

## Discussion

This study introduces a Euclidean Consistency-driven dual-layer fusion framework for UAV flight parameter optimization. The framework integrates a linear statistical layer and a nonlinear learning layer, dynamically regulated by the ECI. This design unifies statistical interpretability with adaptive nonlinear modeling. The analysis identifies optimal parameters as a flight height of 20–25 m, a forward overlap of 80–85%, and a side overlap of 70–75%. Applying these parameters effectively reduces modeling errors. These ranges align with findings in forestry and agricultural studies, where similar combinations are reported to minimize errors [19,21]. In this study, the minimization of elevation error is proposed under 80–85% forward overlap and 70–75% side overlap. This finding matches the 15% reduction in elevation error observed by Grybas and Congalton under 70–85% overlap conditions in forest terrains [11].

However, key differences emerge when comparing our findings to prior work. First, traffic accident reconstruction requires higher overlap to meet strict accuracy standards. Agricultural studies often favor computational efficiency over maximum overlap [17]. Second, horizontal error increases when side overlap exceeds 80%. This contrasts with the steady accuracy gains reported in heterogeneous environments [19]. The difference arises from the homogeneous texture of road surfaces. Excessive overlap can amplify mismatches in repetitive features, an issue less common in complex terrains. Furthermore, this study explicitly quantifies distortion anomaly as a novel accuracy metric-an approach not commonly adopted in earlier research.

In terms of model comparison, the proposed dual-layer fusion framework is benchmarked against Genetic Algorithm (GA) and Response Surface Methodology (RSM). Although no statistically significant difference in predictive accuracy is observed between the fusion model and RSM (0.5085 vs 0.5124, P = 0.974), the proposed framework demonstrates notable advantages in interpretability and parameter stability. Compared with the broad solution space of GA and the assumption-sensitive behavior of RSM to model assumptions under small-sample conditions, the proposed method integrates a linear interpretable module with a nonlinear learning layer. This design enables both transparent interpretation of parameter effects and effective capture of complex nonlinear relationships. Under the Euclidean Consistency constraint (ECI = 0.9133), this framework further yields compact and stable parameter intervals (flight altitude 20–25 m, forward overlap 80–85%, side overlap 70–75%). Therefore, the primary contribution of the proposed framework lies not in improving predictive accuracy, but in providing a theoretically grounded, interpretable, and structurally robust parameter optimization strategy, which is particularly suitable for small-sample conditions.

Given the limited sample size (n = 27) and the inherent complexity of the dual-layer fusion structure, there exists a potential risk of overfitting. To mitigate this risk, several design strategies are adopted. The nonlinear layer is implemented using SVR, which is suitable for small-sample nonlinear regression tasks and incorporates structural risk minimization to control model complexity. In addition, the hyperparameter search space is deliberately constrained, and low-dimensional input features are adopted to further restrict model flexibility and reduce the likelihood of overfitting. Furthermore, the ECI functions as an implicit regularization mechanism that penalizes discrepancies between the linear statistical structure and nonlinear learning outputs, thereby enhancing model stability. The stability of the proposed framework is also supported by the consistency of optimal parameter ranges and overall performance trends across different experimental configurations. The relatively narrow parameter intervals further suggest that the model is less sensitive to data perturbations and exhibits reasonable robustness under the available data conditions.

## Limitations and future work

This study has several limitations. First, the framework is developed using a small-sample dataset (27 flight configurations) from a single experimental site, which limits statistical power and affected the stability and generalizability of the results. For instance, the relatively large variance observed in cross-validated R^2^ for elevation error (RCV^2^  = 0.31 ± 0.36) suggests sensitivity to data partitioning under small-sample conditions. Similarly, some correlations do not reach statistical significance (e.g., forward overlap vs. elevation error, p = 0.144), which, to some extent, reflects limited statistical power rather than the absence of a potential association between variables.

Second, the experiments are conducted using a single UAV platform with a fixed sensor configuration under controlled environmental conditions and primarily static accident scenes. As a result, the identified optimal parameter ranges are influenced by specific hardware performance and scene settings.

Third, no empirical comparison has been made between the chosen SVR-based nonlinear layer and other baseline regression models, such as linear regression, decision trees, or random forest. Consequently, the extent to which the observed performance is attributable to the dual-layer fusion framework versus the specific choice of SVR remains unquantified.

Finally, the validation experiments based on real-world accident cases demonstrate that the proposed framework achieves stable reconstruction performance in practical scenarios, thereby verifying the reliability of the recommended parameter ranges. However, the applicability of this framework in more complex and heterogeneous environments (e.g., varying terrain, surface textures, and environmental conditions), still requires further investigation.

Future research will focus on expanding the dataset size and incorporating more diverse application scenarios, including varying meteorological conditions and multiple UAV platforms, to further evaluate the robustness of the proposed framework. In addition, comparisons with other commonly used regression models will be conducted to assess its generalization capability. These efforts will enhance the framework’s generalization capability and practical applicability in complex real-world environments.

## Conclusions

This study investigates how key flight parameters affect the accuracy of 3D accident scene reconstruction using UAV photogrammetry. A Euclidean Consistency-driven dual-layer information fusion framework is proposed to optimize these parameters. The framework combines a statistically interpretable linear layer with an adaptive nonlinear learning layer.

The experiments demonstrate that flight altitude, forward overlap, and side overlap have distinct but coupled effects on reconstruction accuracy. The optimal configuration is determined as a flight altitude of 20–25 m, a forward overlap of 80–85%, and a side overlap of 70–75%. This parameter set yields centimeter-level precision and minimized geometric distortion.

Within the tested conditions, the proposed method provides a quantitatively interpretable and operationally stable approach for UAV-based 3D reconstruction. This study offers a foundation for the standardization of UAV flight parameters in accident investigation.

## Supporting information

S1 File Dual-layer information fusion code. (PY)

S2 File Linear information layer code. (PY)

S3 File Nonlinear information layer code. (PY)

S4 File Summary of experimental data. (XLSX)
